# The *Haemophilus influenzae* HMW1C Protein Is a Glycosyltransferase That Transfers Hexose Residues to Asparagine Sites in the HMW1 Adhesin

**DOI:** 10.1371/journal.ppat.1000919

**Published:** 2010-05-27

**Authors:** Susan Grass, Cheryl F. Lichti, R. Reid Townsend, Julia Gross, Joseph W. St. Geme

**Affiliations:** 1 Department of Pediatrics, Duke University Medical Center, Durham, North Carolina, United States of America; 2 Department of Medicine Washington University School of Medicine, St. Louis, Missouri, United States of America; 3 Department of Cell Biology and Physiology, Washington University School of Medicine, St. Louis, Missouri, United States of America; 4 Department of Molecular Genetics & Microbiology, Duke University Medical Center, Durham, North Carolina, United States of America; The Rockefeller University, United States of America

## Abstract

The *Haemophilus influenzae* HMW1 adhesin is a high-molecular weight protein that is secreted by the bacterial two-partner secretion pathway and mediates adherence to respiratory epithelium, an essential early step in the pathogenesis of *H. influenzae* disease. In recent work, we discovered that HMW1 is a glycoprotein and undergoes N-linked glycosylation at multiple asparagine residues with simple hexose units rather than N-acetylated hexose units, revealing an unusual N-glycosidic linkage and suggesting a new glycosyltransferase activity. Glycosylation protects HMW1 against premature degradation during the process of secretion and facilitates HMW1 tethering to the bacterial surface, a prerequisite for HMW1-mediated adherence. In the current study, we establish that the enzyme responsible for glycosylation of HMW1 is a protein called HMW1C, which is encoded by the *hmw1* gene cluster and shares homology with a group of bacterial proteins that are generally associated with two-partner secretion systems. In addition, we demonstrate that HMW1C is capable of transferring glucose and galactose to HMW1 and is also able to generate hexose-hexose bonds. Our results define a new family of bacterial glycosyltransferases.

## Author Summary

Decoration of proteins with carbohydrates has an important impact on protein function throughout biology and has been recognized increasingly in pathogenic bacteria. *Haemophilus influenzae* is a common cause of both bacterial respiratory tract disease and bacterial invasive disease and initiates infection by colonizing the upper respiratory tract. The *Haemophilus* HMW1 adhesin is a large protein that resides on the bacterial surface and mediates bacterial attachment to respiratory epithelial cells, an essential step in the process of colonization. In recent work, we discovered that HMW1 is decorated at multiple sites with short carbohydrate units that serve to prevent degradation and to stabilize association with the bacterial surface. In the current study we identify the enzyme responsible for adding carbohydrate units at specific sites of HMW1. In addition, we demonstrate that this enzyme is capable of creating both carbohydrate-protein and carbohydrate-carbohydrate bonds. The amino acid sequence of this enzyme is similar to the sequences of proteins in several other bacteria, suggesting a new family of bacterial enzymes capable of creating carbohydrate-protein and carbohydrate-carbohydrate bonds.

## Introduction

Glycosylation of proteins is an essential process that plays an important role in protein structure and function and represents a strategy to fine tune cell-cell recognition and signaling. For a long period of time, glycosylation of proteins was believed to be restricted to eukaryotes. However, in recent years glycoproteins have been identified increasingly in prokaryotes as well, including pathogenic bacteria such as *Pseudomonas aeruginosa*, *Campylobacter spp.*, *Neisseria spp.*, and *E. coli*, among others [1]–[10].

Nonencapsulated (nontypable) *Haemophilus influenzae* is a human specific pathogen that is a common cause of localized respiratory tract and invasive disease and initiates infection by colonizing the upper respiratory tract [11], [12]. Approximately 75–80% of isolates express two related high-molecular weight proteins called HMW1 and HMW2 that mediate high-level adherence to respiratory epithelial cells and facilitate the process of colonization [13], [14]. The HMW1 and HMW2 adhesins are encoded by homologous chromosomal loci that appear to represent a gene duplication event and contain 3 genes, designated *hmw1A, hmw1B,* and *hmw1C* and *hmw2A, hmw2B,* and *hmw2C*, respectively [15], [16].

**Figure 1:**
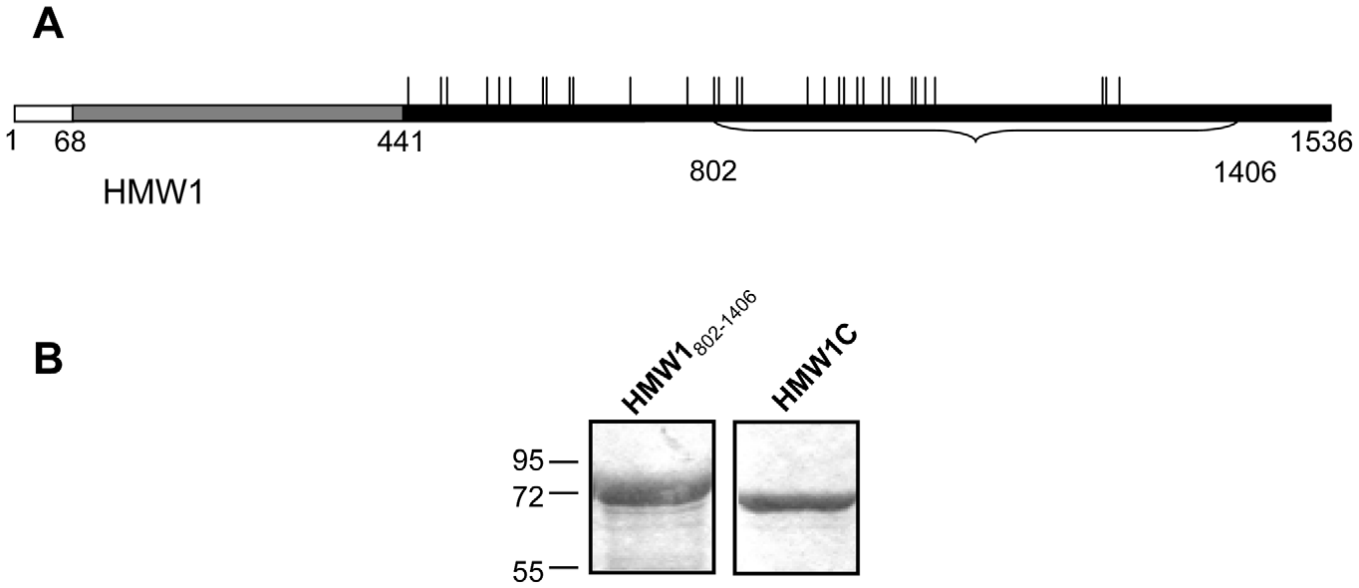
Purified proteins for examination of glycosylation of HMW1. Panel A shows a schematic of the HMW1 pre-pro-protein. The white bar represents the signal peptide, corresponding to amino acids 1–68. The gray bar represents the pro-piece, corresponding to amino acids 69–441. The black bar represents the mature protein, corresponding to amino acids 442–1536. The vertical ticks above the black bar represent sites of N-linked glycosylation. The portion of HMW1 that was used as the acceptor protein for *in vitro* glycosyltransferase assays is highlighted by the bracket and corresponds to amino acids 802–1406 and contains 18 sites of glycosylation. Panel B shows Coomassie blue-strained gels of purified strep-tagged HMW1_802–1406_ and purified HAT-tagged HMW1C.

HMW1 and HMW2 are synthesized as pre-pro-proteins (Figure 1A) and are secreted by the two-partner secretion system [17]–[19]. Amino acids 1–68 represent an atypical signal peptide and direct the pre-pro-proteins to the Sec apparatus, where they are cleaved by signal peptidase I [18]. The resulting pro-proteins are targeted to the HMW1B and HMW2B outer membrane translocators and undergo cleavage between amino acids 441 and 442, removing the pro-pieces and generating mature species that are 125 kDa and 120 kDa, respectively [18]–[21] (Figure 1A). Following translocation across the outer membrane, mature HMW1 and HMW2 remain non-covalently associated with the bacterial surface [18], [19].

In recent work, we demonstrated that HMW1 is a glycoprotein and undergoes glycosylation in the cytoplasm in a process that is dependent upon HMW1C [22]. Functional analyses revealed that glycosylation of HMW1 protects against premature degradation, analogous to some eukaryotic proteins [22]. In addition, glycosylation appears to influence HMW1 tethering to the bacterial surface, a prerequisite for HMW1-mediated adherence [22]. Based on carbohydrate composition analysis of purified HMW1 using gas chromatography and combined gas chromatography-mass spectrometry, the modifying sugars include glucose, galactose, and possibly small amounts of mannose [22]. Analysis of HMW1 proteolytic fragments by mass spectrometry identified 31 sites of modification [23]. All of the modified sites were asparagine residues, in all except one case within the conventional sequence motif for eukaryotic N-linked glycosylation, namely NX(S/T) where X is any residue except for proline [23]. LC-MS/MS analysis, accurate mass measurement, and deuterium replacement studies established that the modifying glycan structures were mono-hexose or di-hexose units rather than N-acetylated hexosamine units that comprise the di-N-diacetyl chitobiose core of eukaryotic and many bacterial asparagine-linked glycans. These results suggested a novel N-linked carbohydrate-peptide transferase activity that does not require assembly of the monosaccharide units onto a lipid-linked intermediate [23].

In the present study, we studied the enzymatic mechanism responsible for the glycosylation of asparagine residues in HMW1. We found that the HMW1C protein encoded in the *hmw1* gene cluster is capable of transferring glucose and galactose to the HMW1 adhesin. In addition, HMW1C is capable of generating hexose-hexose linkages.

## Results

### HMW1C is a glycosyltransferase

In earlier work we found that insertional inactivation of hmw1C in H. influenzae strain Rd-HMW1 resulted in a loss of glycosylation of HMW1 [22], suggesting that HMW1C participates in the process of glycosylation. Further analysis revealed that amino acids 386–439 in HMW1C share 40–41% identity and 51–65% similarity with a domain conserved in a family of eukaryotic O-GlcNAc transferases, including human O-GlcNAc transferase, rat O-GlcNAc transferase, and a plant protein called Spy [22], raising the possibility that HMW1C is a glycosyltransferase.

**Figure 2:**
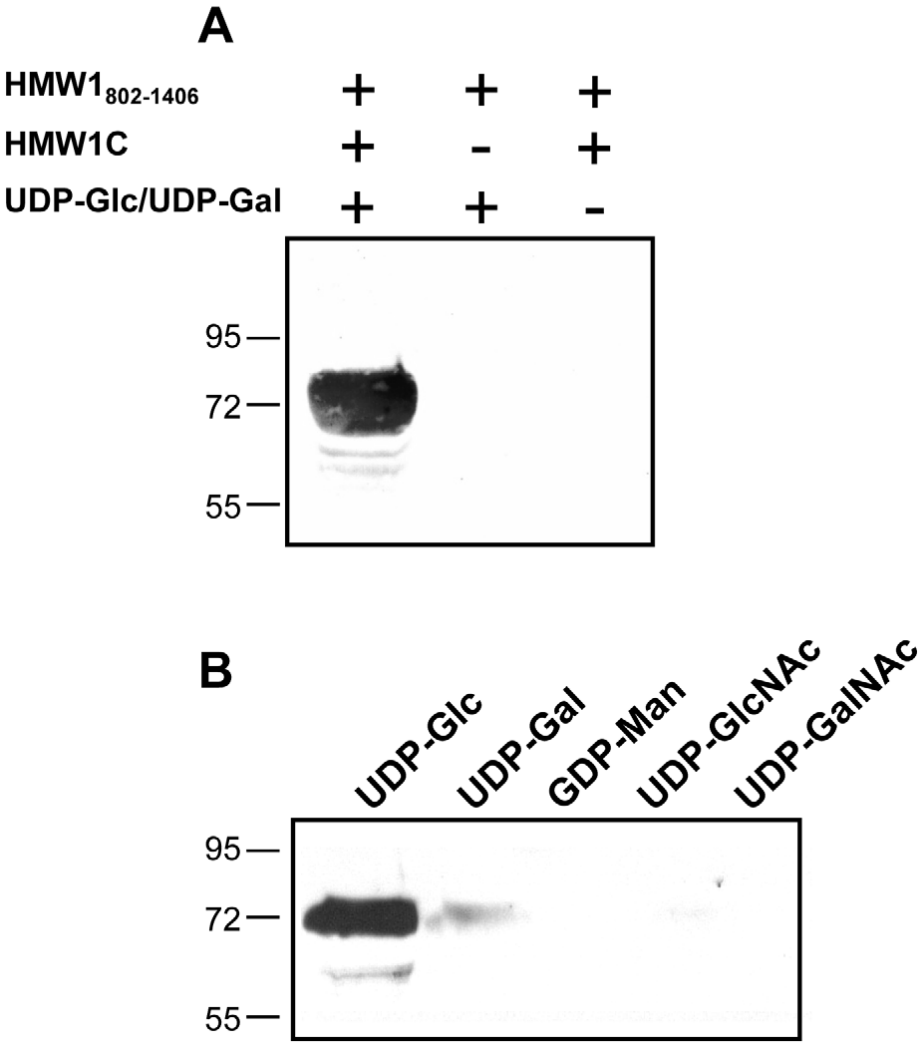
*In vitro* transferase assays, examining samples by SDS-PAGE and detecting glycosylation with DIG-glycan reagents. Panel A shows results with various combinations of purified HMW1_802–1406_, purified HMW1C, and UDP-α-D-glucose plus UDP-α-D-galactose. Panel B shows results with purified HMW1C, purified HMW1_802–1406_, and either UDP-α-D-glucose, UDP-α-D-galactose, GDP-α-D-mannose, UDP-α-D-N-Acetylglucosamine, or UPD-α-D-N-Acetylgalactosamine.

To address the possibility that HMW1C is the glycosyltransferase responsible for N-linked glycosylation of HMW1, we purified HAT-tagged HMW1C and Strep-tagged HMW1_802–1406_ (Figure 1B). HMW1_802–1406_ corresponds to just over half of mature HMW1 (HMW1_442–1536_), contains 18 documented N-linked glycosylation sites, and was more amenable to purification than mature HMW1 (Figure 1A). Subsequently, we incubated approximately equimolar quantities of HAT-HMW1C and Strep-HMW1_802–1406_ with both UDP-α-D-glucose and UDP-α-D-galactose at room temperature for 60 minutes, then examined the reaction mixture for reactivity with the DIG-glycan reagents. As shown in Figure 2A, we observed efficient glycosylation of HMW1_802–1406_ that was dependent on both HMW1C and the UDP-hexoses. To extend this result, we performed the same experiment with UDP-α-D-glucose by itself, UDP-α-D-galactose by itself, GDP-α-D-mannose by itself, UDP-α-D-N-Acetylglucosamine by itself, and UPD-α-D-N-Acetylgalactosamine by itself. As shown in Figure 2B, we observed glycosylation with UDP-α-D-glucose alone and UDP-α-D-galactose alone but not with GDP-α-D-mannose, UDP-α-D-N-Acetylglucosamine, or UPD-α-D-N-Acetylgalactosamine alone. To determine whether smaller amounts of HMW1C are associated with appreciable glycosylation of HMW1_802–1406_, we repeated assays with a fixed amount of HMW1_802–1406_, fixed amounts of UDP-α-D-glucose and UDP-α-D-galactose, and dilutions of HMW1C. Based on analysis using DIG-glycan reagents, we observed efficient glycosylation with molar quantities of HMW1C that were less than one-tenth the molar quantity of HMW1_802–1406_ (data not shown).

### LC-MS/MS analysis of HMW1 after in vitro glycosylation demonstrates specificity of glycosylation with glucose versus galactose

To address whether the glycosylation of HMW1_802–1406_ in in vitro reactions mimicked glycosylation of native HMW1 in whole bacteria and to gain further insight into which sugars modify which sites, we repeated reactions with purified Strep-tagged HMW1_802–1406_, purified HAT tagged HMW1C, and UDP-α-D-glucose alone, UDP-α-D-galactose alone, GDP-α-D-mannose alone, or UDP-α-D-glucose plus UDP-α-D-galactose plus GDP-α-D-mannose and then examined the reaction mixtures by LC-MS/MS. As a positive control we examined purified HMW1_802–1406_ recovered from DH5α/pASK-HMW1_802–1406_ + pHMW1C, and as a negative control we examined HMW1_802–1406_ recovered from DH5α/pASK-HMW1_802–1406_ (lacking pHMW1C).

As summarized in Table 1, we detected 10 of the 18 predicted sites of glycosylation and 11 distinct glycopeptides in HMW1_802–1406_, including 10 glycopeptides with a single site of glycosylation and one glycopeptide with two sites of glycosylation (KNITFEGGNITFGSR). Interestingly, of the 10 sites of glycosylation, all were modified in the in vitro reactions with UDP-α-D-glucose alone and with UDP-α-D-glucose plus UDP-α-D-galactose plus GDP-α-D-mannose. In contrast, only 6 of the 10 sites of glycosylation were modified in the in vitro reactions with UDP-α-D-galactose alone. Consistent with our observations using DIG-Glycan reagents, no sites were glycosylated in the in vitro reactions with GDP-α-D-mannose alone.

**Table 1:** Glycopeptides detected in *in vitro* glycosylation assays with HMW1_802–1406_.

Sequence*	observed *m/z*	calculated *m/z*	Reactions yielding glycopeptide†
LTQDLNISGFNK	756.3823	756.383	Glu, Gal, Glu/Gal/Man
TIISGNITNK	611.8281	611.8299	Glu,Glu, Gal, Glu/Gal/Man
KNITFEGGNITFGSR	982.9750	982.976	Glu,Glu, Glu/Gal/Man
NVTVNNNITSHK	751.87476	751.8759	Glu,Glu, Gal, Glu/Gal/Man
AGVDGENSDSDATNNANLTIK	1134.5079	1134.5091	Glu,Glu, Gal, Glu/Gal/Man
AITNFTFNVGGLFDNK	960.4725	960.4729	Glu,Glu, Gal, Glu/Gal/Man
AITNFTFNVGGLFDNK	1041.4988	1041.4993	Glu,Glu, Glu/Gal/Man
NGDLNITNEGSDTEMQIGGDVSQK	1342.5945	1342.5956	134 Glu, Glu/Gal/Man
NLSITTNSSSTYR	803.3851	803.3838	Glu,Glu, Gal, Glu/Gal/Man
NLSITTNSSSTYR	884.4091	884.4102	Glu,Glu, Glu/Gal/Man
EGNLTISSDK	613.2985	613.2933	613.Glu, Glu/Gal/Man

*Underlined residues represent hexosylated sites, and boldfaced residues represent dihexosylated sites.

†Glu represents UDP-α-D-glucose, Gal represents UDP-α-D-galactose, and Glu/Gal/Man represents UDP-α-D-glucose plus UDP-α-D-galactose plus GDP-α-D-mannose.

**Figure 3:**
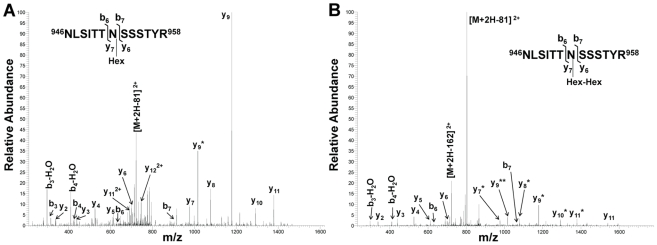
Collision-induced fragmentation spectra from glycosylated peptide NLSITTNSSSTYR (HMW1 amino acids 946–958). Panel A shows the CID spectrum of the glycopeptide that is modified with one hexose unit. Panel B shows the CID spectrum of the glycopeptide that is modified with a di-hexose. The asterisks indicate b and y fragmentation ions that underwent a neutral loss of one (*) or two (**) hexosyl residues.

As demonstrated by the collision-induced fragmentation spectra shown in Figure 3 and Figure S1, the glycopeptide NLSITTNSSSTY (HMW1 amino acids 946–958, with glycosylation at N952) and the glycopeptide AITNFTFNVGGLFDNK (HMW1 amino acids 909–924, with glycosylation at N912) were present in two forms, including one with a mono-hexose at the predicted site of glycosylation and the other with a di-hexose at the predicted site of glycosylation. The forms containing a mono-hexose were detected in the in vitro reactions with UDP-α-D-glucose alone, UDP-α-D-galactose alone, and UDP-α-D-glucose plus UDP-α-D-galactose plus GDP-α-D-mannose, while the forms containing a di-hexose were detected only in the in vitro reactions with UDP-α-D-glucose alone and with UDP-α-D-glucose plus UDP-α-D-galactose plus GDP-α-D-mannose, suggesting that glucose must be the first hexose linked to asparagine in the glycopeptides containing di-hexose modification.

Together these findings demonstrate that the HMW1C protein is a glycosyltransferase and has a novel activity capable of transferring glucose and galactose to asparagine residues in HMW1 and creating hexose-hexose bonds. In addition, they demonstrate that the di-hexosylated sites at N951 and N912 are initially modified with a glucose monosaccharide.

### Inactivation of galU eliminates glycosylation of HMW1

**Figure 4:**
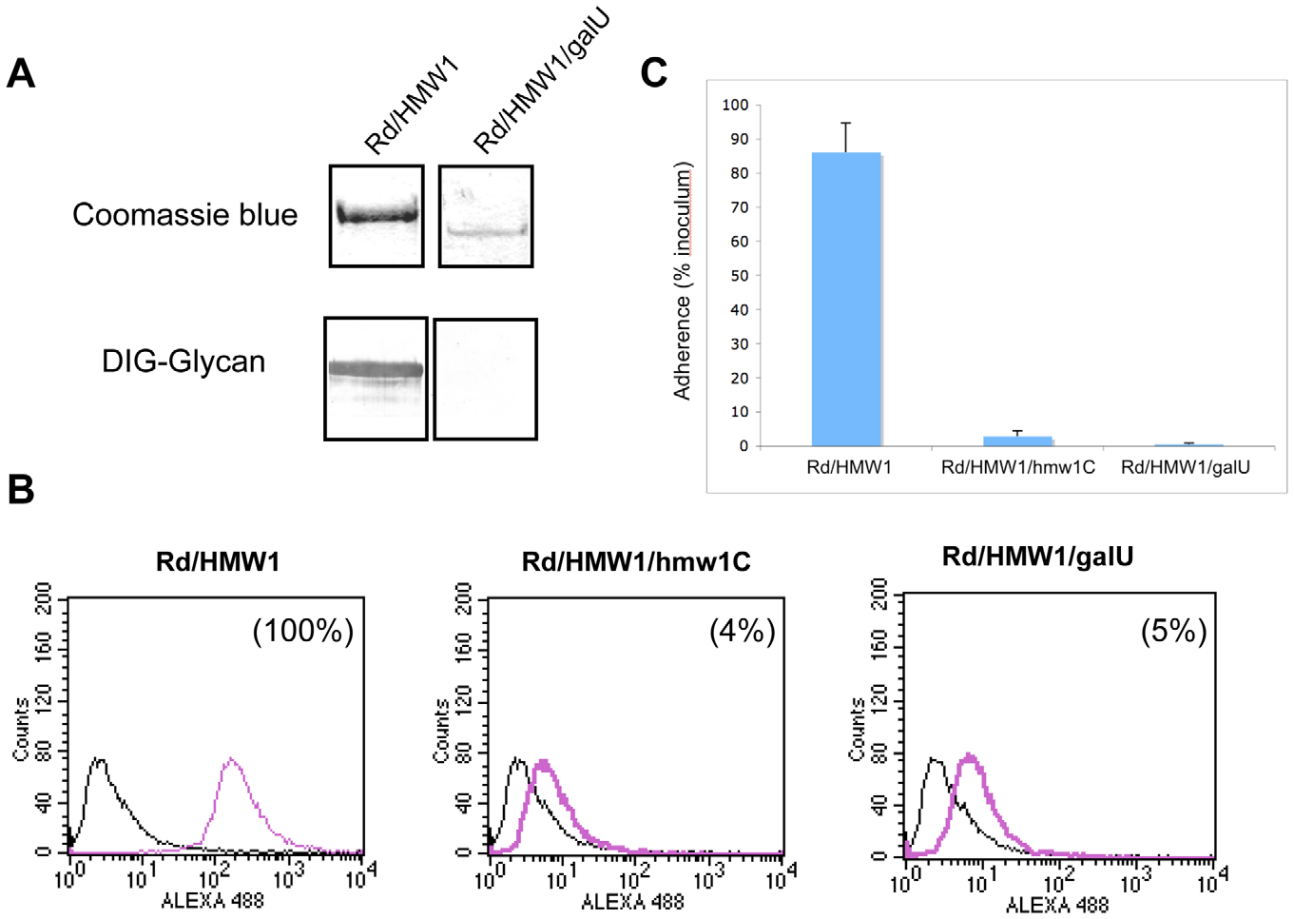
Effect of inactivation of the *galU* gene. Panel A shows purified HMW1 recovered from strain Rd-HMW1 and Rd-HMW1/galU. With strain Rd-HMW1, HMW1 was recovered from the surface of whole bacteria. With strain Rd-HMW1/galU, HMW1 was recovered from the culture supernatant and concentrated, reflecting the fact that HMW1 is no longer surface associated in this strain. Purified protein was resolved by SDS-PAGE and was then either stained with Coomassie blue (upper gel) or examined for glycosylation using DIG-glycan reagents (lower gel). Panel B shows flow cytometry results with strains Rd-HMW1, Rd-HMW1/hmw1C, and Rd-HMW1/galU using antiserum GP85 against HMW1 to detect surface-associated HMW1. The number in the upper right corner of the plots of Rd-HMW1/hmw1C and Rd-HMW1/galU represents the percentage of surface-associated HMW1 in strain Rd-HMW1. Panel C shows *in vitro* adherence results comparing adherence by Rd-HMW1, Rd-HMW1/hmw1C, and Rd-HMW1/galU to Chang epithelial cells. Bars and error bars represent mean and standard deviations of measurements performed in triplicate.

To extend our understanding of glycosylation of HMW1 and confirm our observation that HMW1 is modified with glucose and galactose in *in vitro* glycosylation assays, we examined the effect of insertional inactivation of *galU* (open reading frame HI0812 in strain Rd) on glycosylation of HMW1 in strain Rd-HMW1. The *galU* gene encodes glucose-1-phosphate uridyl transferase, which converts glucose-1-phosphate to UDP-glucose (Figure S2). UDP-glucose in turn can be converted directly to UDP-galactose by GalE (UDP Gal-4-epimerase) or can serve as the donor of UDP for conversion of galactose-1-phosphate to UDP-galactose. In assessing the effect of inactivation of *galU*, we incubated Rd-HMW1/galU in supplemented brain heart infusion broth [24], which contains glucose as the primary carbon source. Interestingly, inactivation of *galU* mimicked the effect of inactivation of *hmw1C* described in our earlier work [22], eliminating HMW1 glycosylation as assessed by DIG-glycan blots (Figure 4A), virtually eliminating HMW1 tethering to the bacterial surface (Figure 4B), and abolishing HMW1-mediated adherence (Figure 4C).

Consistent with our *in vitro* glycosyltransferase assays with purified HMW1_802–1406_ and HMW1C, these results indicate that UDP-glucose is required for glycosylation of HMW1 in *H. influenzae* under standard growth conditions in supplemented brain heart infusion broth.

## Discussion

In this study, we found that the *H. influenzae* HMW1C protein encoded in the *hmw1* gene cluster is a glycosyltransferase and is capable of transferring glucose and galactose to asparagine residues in the HMW1 adhesin, providing the first example of a glycosyltransferase that transfers hexose units rather than N-acetylated amino sugars to asparagine residues in protein targets. Further analysis revealed that HMW1C is capable of creating both hexose-asparagine and hexose-hexose linkages, suggesting multi-functionality as a glycosyltransferase.

All previously reported carbohydrate modification of asparagine residues in proteins in Eukarya and Bacteria involve the *en bloc* transfer of oligosaccharides from a lipid-linked intermediate by an oligosaccharyltransferase complex [25]. In Archaea, the mechanisms of N-glycosylation are less well understood. Glycosylation of asparagine residues with a trisaccharide moiety in the flagellin and S-layer proteins of *Methanococcus voltae* has been proposed to proceed via a lipid-linked intermediate [26]. More recently it has been shown that hexose units are attached directly to asparagine residues in an S layer glycoprotein of *Haloferax volcanii* [27]. A pentasaccharide with the structure Hex-X-hexuronic acid-HexA-HexA-Hex-peptide was identified at two glycosylation sites. Interestingly, these two sites were different from the conventional N-glycosylation sequence motif observed in eukaryotes and in HMW1. It is currently unclear whether the *H. volcanii* Hex-Asn linkage is formed from a lipid-linked intermediate or via activated monosaccharides as we have found with HMW1 and HMW1C.

In earlier work, we performed carbohydrate composition analysis on purified HMW1 and detected glucose, galactose, and small amounts of mannose [22]. Given the potential for contaminating sugars to be detected in this analysis, we were uncertain as to whether mannose was truly present as a modifying sugar in HMW1, especially given that it accounted for only 2.5–3% of the total carbohydrate [22]. Our analysis in the current study argues that mannose is not present in HMW1. In particular, in *in vitro* glycosyltransferase assays using purified HMW1_802–1406_, HMW1C, and GDP-α-D-mannose, we were unable to detect modification of HMW1_802–1406_ using either DIG-Glycan reagents or LC-MS/MS.

Based on assessment of the 10 glycopeptides that we detected in our *in vitro* glycosylation assays with HMW1_802–1406_, which corresponds to just over half of mature HMW1, we observed that HMW1C transfers glucose to all glyscosylated asparagines and transfers galactose to only a subset of glyscosylated asparagines. All of these glycosylation sites correspond to the conventional sequence motif of N-linked glycans, namely NX(S/T), with X being any amino acid except proline. Examination of the primary amino acid sequence of the sites that are modified only with glucose and the sites that are modified with either glucose or galactose in *in vitro* assays reveals no apparent distinction, suggesting that factors beyond the amino acid sequence influence the specificity or potentially the efficiency of glycosylation. This observation is consistent with the fact that only a fraction of conventional sequences motifs are glycosylated in HMW1 purified from *H. influenzae* [23].

Further analysis of the glycopeptides detected after *in vitro* glycosylation revealed two peptides that were modified with a di-hexose. Interestingly, in both cases the glycopeptides were detected only in the reactions performed with UDP-α-D-glucose alone and with UDP-α-D-glucose plus UDP-α-D-galactose plus GDP-α-D-mannose, indicating modification with UDP-α-D-glucose. In contrast, the corresponding glycopeptides containing a single hexose at the asparagines in question were detected in the reactions performed with UDP-α-D-glucose alone, with UDP-α-D-galactose alone, and with UDP-α-D-glucose plus UDP-α-D-galactose plus GDP-α-D-mannose, indicating modification with either glucose or galactose. Considered together, these results suggest that glucose must be linked to asparagine in the glycopeptides containing di-hexose modification. At this point, it is unclear whether the di-hexose is generated prior to modification of the acceptor asparagine residue or whether instead a single hexose is linked to the target asparagine and then a second hexose is linked to the first hexose, although the conventional interpretation is that the hexose is added to the protein and then the chain is extended. In either event, it appears that HMW1C is responsible for creating the hexose-hexose bond.

Interestingly, homology analysis reveals 42–68% identity and 58–83% similarity between the full-length HMW1C sequence and proteins in a number of other gram-negative bacterial pathogens, including the enterotoxigenic *E. coli* (ETEC) EtpC protein and predicted proteins in *Yersinia pseudotuberculosis, Y. enterocolitica, Y. pestis, H. ducreyi, Actinobacillus pleuropneumoniae, Mannheimia spp., Xanthomonas spp.*, and *Burkholderia spp*, among others (Table S1). In ETEC, *Y. pseudotuberculosis, Y. enterocolitica,* and *Y. pestis,* these homologs are encoded by genes that are adjacent to known or predicted two-partner secretion loci. The *H. ducreyi, Mannheimia succiniciproducens,* and *Burkholderia xenovorans* genomes contain genes that encode predicted two-partner secretion proteins as potential targets for the HMW1C homologs, although these genes are in unlinked locations. The ETEC EtpC protein is encoded by a two-partner secretion locus called *etpBAC* and has been shown to be required for glycosylation of the EtpA adhesin, a high-molecular weight protein that has a predicted molecular mass of ∼177 kDa and promotes adherence to intestinal epithelial cells and colonization of the intestine in mice [28], [29]. These observations suggest that that there is a family of bacterial HMW1C-like proteins with glycosyltransferase activity.

To summarize, in eukaryotes N-linked glycosylation occurs in the endoplasmic reticulum and involves an oligosaccharyltransferase that catalyzes the transfer of the oligosaccharide from the lipid donor dolichylpyrophosphate to the acceptor protein. Similarly, in bacteria, N-glycosylation generally occurs in the periplasm and involves an oligosaccharyltransferase that transfers the glycan structure from a lipid donor to the acceptor protein. In contrast, in the case of the *H. influenzae* HMW1 adhesin, N-linked glycosylation occurs in the cytoplasm and involves direct transfer of hexose units to the acceptor protein by HMW1C, with no requirement for a lipid donor. In this study, we have established that the *H. influenzae* HMW1C protein is a multi-functional enzyme that is capable of transferring glucose and galactose to asparagine residues in selected conventional N-linked sequence motifs in HMW1 and is also capable of creating hexose-hexose linkages. Based on homology analysis, it is likely that a variety of other bacteria possess HMW1C-like proteins with similar enzymatic activity. In future work, we will examine whether these HMW1C-like proteins are identical to HMW1C in terms of the glycan units that they transfer and the acceptor protein sequence motifs that they recognize.

## Materials and Methods

### Bacterial strains and plasmids

The strains and plasmids used in this study are listed in Table 2. *H. influenzae* strain Rd-HMW1 is a derivative of strain Rd that contains the intact *hmw1* locus and expresses fully functional HMW1 [22]. *H. influenzae* strain Rd-HMW1/hmw1C is a derivative of strain Rd-HMW1 that contains an insertionally inactivated *hmw1C* gene [22]. The *H. influenzae* Rd-HMW1 derivative harboring a kanamycin cassette in *galU* was constructed by transforming competent Rd-HMW1 with genomic DNA recovered from Rd*galU* and selecting for kanamycin resistance [30].

**Table 2:** Strains and plasmids used in this study.

Strains	Description	Source/Reference
*H. influenzae*		
Rd	Non-adherent non-encapsulated laboratory strain, formerly type d	
Rd-HMW1	Derivative of Rd containing *hmw1* locus and expressing HMW1	22
Rd-HMW1/hmw1C	Derivative of Rd-HMW1 with Kan^R^ cassette in *hmw1C*	22
Rd-HMW1/galU	Derivative of Rd-HMW1 with Kan^R^ cassette in *galU*	This study
*E. coli*		
DH5α	*E. coli* F- Ф80d*lacZ*ΔM15 Δ (*lacZYA-argF*) *U169 deoR recA1 endA1 hsdR17*(rK-mK+) *phoA supE441 thi-1 gyrA96 relA1*	28

Plasmids	Description	Source
pUC18	Cloning vector	
pUC4K	Cloning vector	Pharmacia
pASK-IBA12	Cloning vector	IBA, BioTAGnology
pASK-HMW1_802–1406_	Derivative of pASK-IBA12 encoding HMW1_802–1406_ with an N-terminal streptactin tag	This study
pHAT10	Cloning vector	Clontech
pHAT-HMW1C	Derivative of pHAT10 encoding HMW1C with an N-terminal HAT epitope	This study

In order to overexpress HMW1_802–1406_ with a Strep tag at the N terminus, the fragment encoding HMW1_802–1406_ was amplified by PCR from pHMW1-14 using a 5′ primer that incorporated a BamHI site and a 3′ primer that incorporated a SalI site. The PCR amplicon was digested with BamI and SalI and then ligated into BamHI-SalI-digested pASK-IBA12 (IBA, BioTAGnology), creating pASK-HMW1_802–1406_.

In order to overexpress the HMW1C protein with a HAT epitope at the N terminus, the *hmw1C* gene was amplified by PCR from pHMW1-14 using a 5′ primer that incorporated a BamHI site and a 3′ primer that incorporated an EcoRI site. The PCR amplicon was digested with BamHI and EcoRI and then ligated into BamHI-EcoRI-digested pHAT10 (Clontech), creating pHAT-HMW1C.

### Transformation and mutagenesis

Plasmids were introduced into *E. coli* by chemical transformation [31]. DNA was introduced into *H. influenzae* using the MIV method of transformation described by Herriott et al. [32]. Transformants were selected by plating on agar containing kanamycin, and mutations were confirmed by PCR analysis using primers that anneal to regions flanking the target gene.

### Protein purification

To purify HMW1_802–1406_, *E. coli* strain DH5α/pASK-HMW1_802–1406_ was grown at 37°C to an OD_600_ of 0.7, then induced for 2 hrs with the addition of 100 µg/ml of anhydro-tetracycline (Sigma). Cells were harvested, resuspended in 100 mM Tris pH 8.0, 150 mM NaCl with Complete Mini protease inhibitor (Roche), and lysed by sonication. Insoluble material was removed by centrifugation at 12,500 × g for 30 min. The supernatant was loaded onto a Strep-Tactin Superflow cartridge and eluted according to the manufacturer's instructions (IBA, BioTAGnology). Eluted fractions were analyzed for purity by SDS-PAGE and were pooled. To purify HMW1C, *E. coli* strain DH5α/pHAT-HMW1C was grown at 37°C overnight. Cells were recovered, resuspended in 50 mM sodium phosphate buffer pH 7.0, 300 mM NaCl (bufferA), and lysed by sonication. Insoluble material was removed by centrifugation at 12,500 × g for 30 min. The supernatant was loaded onto a 1 ml Talon column (Clontech) and eluted with a gradient of 0 to 300 mM imidazole in Buffer A. Fractions were analyzed for purity by SDS-PAGE and were pooled.

### Glycosyltransferase assay

In standard *in vitro* glycosyltransferase assays, 1.5 µg (23 pmole) of purified HMW1_802–1406_ was combined with a mixture containing 20 µl of 50 mM UDP-α-D-glucose, 50 mM UDP-α-D-galactose, 50 mM GDP-α-D-mannose, 50 mM UDP-α-D-N-Acetylglucosamine, or 50 mM UDP-α-D-N-acetylgalactosamine (Calbiochem) either as individual sugars or as mixtures. The reactions were initiated with addition of 1.5 µg (21 pmole) of purified HMW1C in a final volume of 150 µl in 25 mM Tris pH 7.2, 150 mM NaCl. Samples were incubated for 60 minutes at room temperature and then further incubated at 4°C overnight.

### Carbohydrate detection

To detect protein glycosylation, DIG Glycan reagents (Roche) were employed. Use of these reagents is based on the oxidation of hydroxyl groups in carbohydrates to aldehydes either in solution or bound to nitrocellulose membranes. Digoxigenin is then covalently linked to the aldehyde groups, and an anti-digoxigenin alkaline-phosphatase conjugated agent is used for detection of labeled carbohydrates.

### Flow cytometry

FACS analysis was performed by the Duke University Medical Center Cancer Research Center Flow Cytometry Shared Resource Center using a Becton Dickinson FACS Calibur instrument at a wavelength of 488 nm. Bacterial suspensions were fixed with 1% formaldehyde in PBS at room temperature for 30 min. After washing once with Tris buffered saline (TBS), bacteria were resuspended in 1 ml of TBS, 50 mM EDTA, 0.1% bovine serum albumin, and a 1∶1000 dilution of guinea pig antiserum GP85 directed against HMW1 [33] and were incubated with gentle rocking at room temperature for 1 hr. Samples were then centrifuged, washed twice with PBS, and resuspended in 200 µl of PBS, 0.1% bovine serum albumin, and a 1∶200 dilution of Alexa Fluor488 anti-guinea pig antibody (Molecular Probes). Samples were incubated with gentle rocking at room temperature for 1 hr. After two additional washes with PBS, bacterial pellets were re-suspended in 1 ml of PBS and were then analyzed. Data were analyzed with CELLQUEST software (Becton Dickinson). To quantify histograms, markers were drawn on plots, and positive events within the markers were determined as a percentage of the positive control (set at 100%).

### Adherence assays

Adherence assays were performed with Chang epithelial cells (human conjunctiva; ATCC CCL 20.2) (Wong-Kilbourne derivative clone 1-5c-4) as described previously [16]. Percent adherence was calculated by dividing the number of adherent colony-forming units by the number of inoculated colony-forming units. All strains were examined in triplicate, and each assay was repeated at least two times.

### Protein analysis

Whole cell sonicates were prepared by suspending bacterial pellets in 10 mM HEPES, pH 7.4 and sonicating to clarity. Proteins were resolved by SDS-PAGE using 10% polyacrylamide gels. Western blots were performed using guinea pig antiserum GP85 against the HMW1 protein [30].

### Protein digestion and peptide preparation

Samples were precipitated using the 2D protein clean up kit (GE Healthcare) according to the manufacturer's instructions. Bovine serum albumin (100 ng) was added to each sample as an internal standard. Pellets were dissolved in 40 µl 9 M urea and aliquoted into 0.5 ml microfuge tubes. Samples (20 µl in 9 M urea) were reduced with 5 mM TCEP at pH 8.0 at room temperature for 30 min and were alkylated with 10 mM iodoacetamide (Bio-Rad) in the dark at room temperature for 30 min. TCEP and iodoacetamide were quenched with 5 mM dithiothreitol (DTT) at room temperature for 10 min. The reduced and alkylated proteins were digested with 1 µg of endoproteinase Lys-C (Roche) at 37°C overnight. Samples were diluted with 64 µl H_2_O to reduce the concentration of urea to 2 M and were then digested with 4 µg trypsin (Sigma) at 37°C overnight. Peptides were acidified with 5.5 µl formic acid (Sigma) and extracted 6 times with 10–200 µl NuTip porous graphite carbon wedge tips (Glygen) according to the manufacturer's directions and were then eluted into 1.5 ml autosampler vials with 60% acetonitrile (Burdick & Jackson) in 0.1% formic acid. The peptide digests were evaluated for quality and detergent contaminants using MALDI-TOF/TOF [34] prior to LC-MS analysis. For MALDI-TOF/TOF analysis, the peptide sample (0.5 µl) was mixed with an equal volume of MALDI matrix solution (Agilent Technologies) prior to spotting. For nano-LC-FTICR-MS analysis, the peptide sample was dried and immediately dissolved in 10 µl aqueous acetonitrile/formic acid (1%/1%).

### Mass spectrometry

The complex mixtures of peptides and glycopeptides from HMW1_802–1406_ were analyzed using high-resolution nano-LC-MS on a hybrid mass spectrometer consisting of a linear quadrupole ion-trap and an Orbitrap (LTQ-Orbitrap XL, Thermo-Fisher). The liquid chromatographs were nanoflow HPLC systems (NanoLC-1Dplus™ and NanoLC-Ultra™) that were interfaced to the mass spectrometer with a nanospray source (PicoView PV550; New Objective). The in-house packed LC column (Jupiter C12 Proteo, 4 µm particle size, 90 Å pore size [Phenomenex]) was equilibrated in 98% solvent A (aqueous 0.1% formic acid) and 2% solvent B (acetonitrile containing 0.1% formic acid). The samples (10 µL) were injected from autosampler vials using the LC-systems autosamplers at a flow rate of 1.0 µL/min and were eluted using a segmented linear gradient (250 nL/min) with solvent B: isocratic at 2% B, 0–2 min; 2% B to 40% B, 2–65 min; 40% B to 80% B, 65–70 min; isocratic at 80% B, 70–72 min; 80% B to 2% B, 72–77 min; and isocratic at 2% B, 77–82 min. The survey scans (*m/z* 350–2000) (MS1) were acquired at high resolution (60,000 at *m/z* = 400) in the Orbitrap, and the MS/MS spectra (MS2) were acquired in the linear ion trap at low resolution, both in profile mode. The maximum injection times for the MS1 scan in the Orbitrap and the LTQ were 50 ms and 100 ms, respectively. The automatic gain control targets for the Orbitrap and the LTQ were 2×10^5^ and 3×10^4^, respectively. The MS1 scans were followed by six MS2 events in the linear ion trap with wideband collision activation in the ion trap (parent threshold = 1000; isolation width = 2.0 Da; normalized collision energy  = 30%; activation Q = 0.250; activation time = 30 ms). Dynamic exclusion was used to remove selected precursor ions (−0.25/+1.5 Da) after MS2 acquisition with a repeat count of 2, a repeat duration of 30 s, and a maximum exclusion list size of 200. The following ion source parameters were used: capillary temperature 200 °C, source voltage 2.5 kV, source current 100 µA, and the tube lens at 79 V. The data were acquired using *X*calibur, version 2.0.7 (Thermo-Fisher).

The MS2 spectra were analyzed both by searching a customized protein database that contained the sequences of HMW_802–1406_ and by expert manual interpretation. The exact masses of the glycopeptides and fragmentation ions were calculated using the Molecular Weight Calculator, version 6.45 (http://ncrr.pnl.gov/software/). For database searches, the LC-MS files were processed using MASCOT Distiller (Matrix Science, version 2.3.0.0) with the settings previously described [35]. The resulting MS2 centroided files were used for database searching with MASCOT, version 2.1.6, and the following parameters: enzyme, trypsin; MS tolerance = 10 ppm; MS/MS tolerance = 0.8 Da with a fixed carbamidomethylation of Cys residues and the following variable modifications: Methionine, oxidation; Pyro-glu (N-term); Maximum Missed Cleavages  = 5; and 1+, 2+, and 3+ charge states.

## Supporting Information

Table S1 HMW1C homologs and potential TpsA and TpsB partners.
(0.03 MB DOC)

Figure S1 Collision-induced fragmentation spectra of glycosylated peptide AITNFTFNVGGLFDNK (HMW1 amino acids 909–924). Panel A shows the CID spectrum of the glycopeptide that is modified with one hexose unit, and Panel B shows the CID spectrum of the glycopeptide that is modified with a di-hexosyl moiety. The asterisks indicate b and y fragmentation ions that underwent a neutral loss of one (*) or two (**) hexosyl residues. A prominent ion that is consistent with the neutral loss of a hexosyl unit was observed as the doubly charged species ([M+2H-81]^+2^). In the spectrum from the mono-hexosylated glycopeptide (Panel A), both y ions (y_3_, y_4_, y_5_, y_6_, y_7_, y_8_, y_10_, y_11_, y_12_, and y_13_ at *m/z* 376.3, 523.3, 636.3, 693.3, 750.4, 849.3, 1110.6, 1211.6, 1358.3, and 1634.8, respectively) and b ions (b_4_, b_5_, b_6_, b_8_, b_9_, b_12_, b_13_, b_14_, and b_15_ at *m/z* 562.1, 709.25, 810.3, 1071.6, 1170.4, 1397.4, 1544.7, 1659.5, and 1773.4, respectively) were observed that were consistent with the amino acid sequence and hexosylation at N912 (**y_13_** is the fragment ion modified with a hexose moiety). In the spectrum from the di-hexosylated glycopeptide (panel B), a similar pattern of neutral loss y and b ions was observed. The base peak in this spectrum (*m/z* 960.3) represents loss of a single hexose moiety from the doubly charged parent ion ([M+2H-81]^2+^), and a peak representing loss of both hexose residues ([M+2H-162]^2+^) is also seen (*m/z* 879.3). Several of the hexose-containing y ions and b ions demonstrated neutral loss of a hexosyl residue, with one hexosyl unit remaining on the y ion fragmentation series (y_13_* and y_14_* at *m/z* 1635.0 and 1735.9, respectively) and b ion fragmentation series (b_4_*, b_5_*, b_6_*, b_9_*, and b_12_* at *m/z* 562.3, 709.3, 810.2, 1170.4, and 1397.4, respectively). The deduced amino acid sequence was supported by other unmodified y ions (y_3_, y_4_, y_5_, y_6_, y_7_, y_8_, y_9_, y_10_, and y_11_ at *m/z* 376.1, 523.3, 636.3, 693.4, 750.3, 849.4, 963.3, 1110.5, and 1211.42, respectively).
(0.51 MB TIF)

Figure S2 The pathway for glucose and galactose metabolism in *H. influenzae*. GalU (G-1-P uridylyltransferase) converts glucose-1-phosphate to UDP-glucose, which in turn is converted to UDP-galactose by GalE (UDP-Gal-4-epimerase) and which also serves as a donor of UDP for conversion of galactose-1-phosphate to UDP-galactose.
(0.06 MB TIF)
