# Supplementary material for: The Haemophilus influenzae HMW1C Protein Is a Glycosyltransferase That Transfers Hexose Residues to Asparagine Sites in the HMW1 Adhesin
Source: PLoS Pathog. 2010 May 27;6(5):e1000919. doi: 10.1371/journal.ppat.1000919 (PMC2877744; doi:10.1371/journal.ppat.1000919)
Supplement: Table S1 — HMW1C homologs and potential TpsA and TpsB partners. (0.03 MB DOC) [file ppat.1000919.s001.doc]

Table S1. HMW1C homologs and potential TpsA and TpsB partners

| **Species** | **HMW1C homolog** | **Putative TpsA*** | **Putative TpsB†** |
| --- | --- | --- | --- |
| *Yersinia enterocolitica* | RscC | RscA | RscB |
| *Yersinia pestis KIM* | Y0944 | Y0942 | Y0941 |
| *Yersinia pseudotuberculosis* | YPK_3311 | YPK_3312 | YPK0086 |
| *Haemophilus ducreyi* | HD1895 | HD1327 | HD1326 |
| *Burkolderia xenovorans* | Bxe_A1440 | Bxe_C1127 | Bxe_B0153 |
| *Escherichia coli* (ETEC) | EtpC | EtpA | EtpB |
| *Mannheimia succiniciproducens* | MS2246 | MS1167 | MS1169 |
| *Limnobacter* Sp. MED105 | LMED105_04712 | LMED105_11935 | LMED105_11930 |
| *Xanthomonas campestris* | XXC0866 | XXC1794 | XXC1793 |

* TpsA corresponds to the protein that is secreted through the two-partner secretion pathway via the cognate TpsB protein.

**†** TpsB is the outer membrane translocator protein component of the two-partner secretion pathway.
