# Supplementary figures and images for: The Haemophilus influenzae HMW1C Protein Is a Glycosyltransferase That Transfers Hexose Residues to Asparagine Sites in the HMW1 Adhesin
Source: PLoS Pathog. 2010 May 27;6(5):e1000919. doi: 10.1371/journal.ppat.1000919 (PMC2877744; doi:10.1371/journal.ppat.1000919)

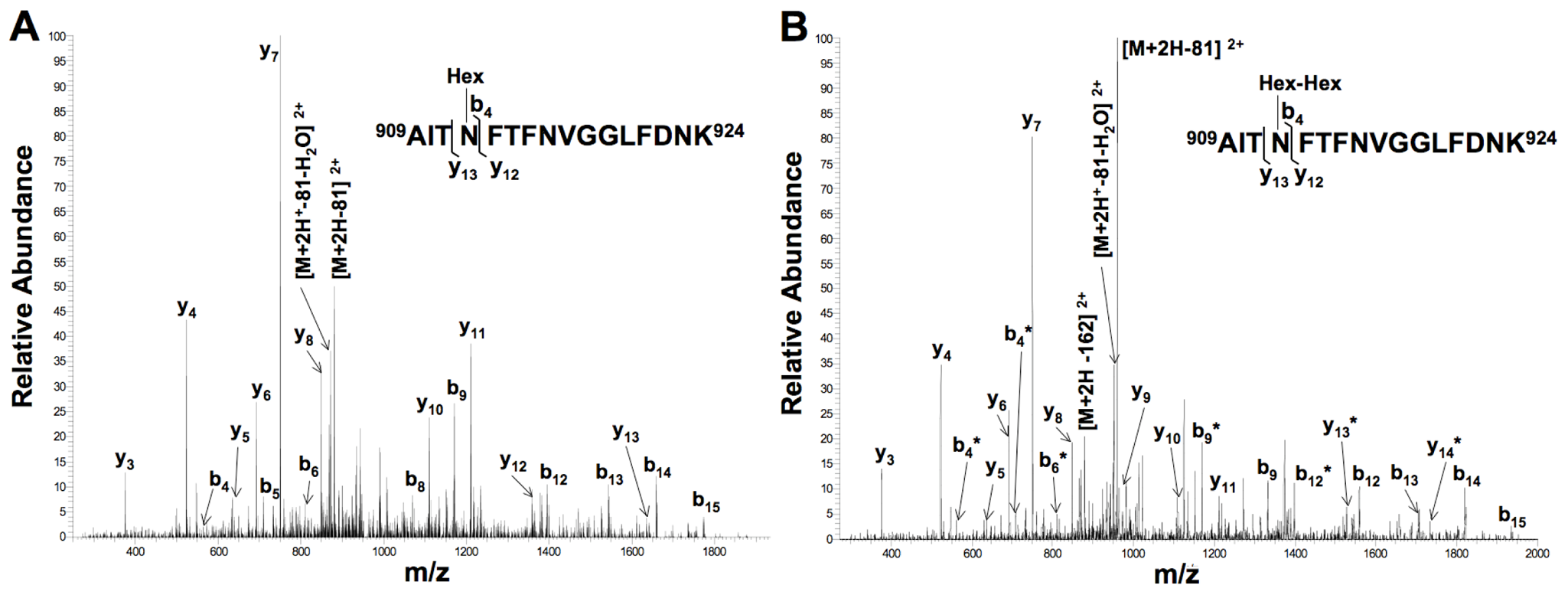

Supplement: Figure S1 — Collision-induced fragmentation spectra of glycosylated peptide AITNFTFNVGGLFDNK (HMW1 amino acids 909–924). Panel A shows the CID spectrum of the glycopeptide that is modified with one hexose unit, and Panel B shows the CID spectrum of the glycopeptide that is modified with a di-hexosyl moiety. The asterisks indicate b and y fragmentation ions that underwent a neutral loss of one (*) or two (**) hexosyl residues. A prominent ion that is consistent with the neutral loss of a hexosyl unit was observed as the doubly charged species ([M+2H-81]+2). In the spectrum from the mono-hexosylated glycopeptide (Panel A), both y ions (y3, y4, y5, y6, y7, y8, y10, y11, y12, and y13 at m/z 376.3, 523.3, 636.3, 693.3, 750.4, 849.3, 1110.6, 1211.6, 1358.3, and 1634.8, respectively) and b ions (b4, b5, b6, b8, b9, b12, b13, b14, and b15 at m/z 562.1, 709.25, 810.3, 1071.6, 1170.4, 1397.4, 1544.7, 1659.5, and 1773.4, respectively) were observed that were consistent with the amino acid sequence and hexosylation at N912 (y13 is the fragment ion modified with a hexose moiety). In the spectrum from the di-hexosylated glycopeptide (panel B), a similar pattern of neutral loss y and b ions was observed. The base peak in this spectrum (m/z 960.3) represents loss of a single hexose moiety from the doubly charged parent ion ([M+2H-81]2+), and a peak representing loss of both hexose residues ([M+2H-162]2+) is also seen (m/z 879.3). Several of the hexose-containing y ions and b ions demonstrated neutral loss of a hexosyl residue, with one hexosyl unit remaining on the y ion fragmentation series (y13* and y14* at m/z 1635.0 and 1735.9, respectively) and b ion fragmentation series (b4*, b5*, b6*, b9*, and b12* at m/z 562.3, 709.3, 810.2, 1170.4, and 1397.4, respectively). The deduced amino acid sequence was supported by other unmodified y ions (y3, y4, y5, y6, y7, y8, y9, y10, and y11 at m/z 376.1, 523.3, 636.3, 693.4, 750.3, 849.4, 963.3, 1110.5, and 1211.42, respectively). (0.51 MB TIF) [file ppat.1000919.s002.tif]

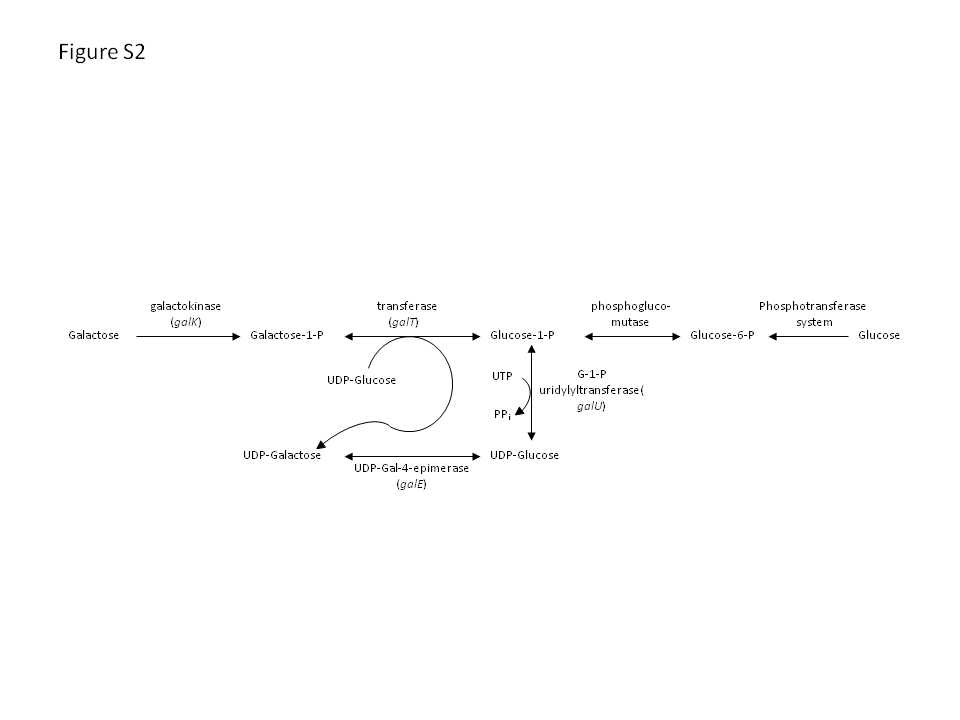

Supplement: Figure S2 — The pathway for glucose and galactose metabolism in H. influenzae. GalU (G-1-P uridylyltransferase) converts glucose-1-phosphate to UDP-glucose, which in turn is converted to UDP-galactose by GalE (UDP-Gal-4-epimerase) and which also serves as a donor of UDP for conversion of galactose-1-phosphate to UDP-galactose. (0.06 MB TIF) [file ppat.1000919.s003.tif]
